# Radiological anatomy of the intracranial vertebral artery in a select South African cohort of patients

**DOI:** 10.1038/s41598-021-91744-9

**Published:** 2021-06-09

**Authors:** B. R. Omotoso, R. Harrichandparsad, K. S. Satyapal, I. G. Moodley, L. Lazarus

**Affiliations:** 1 Discipline of Clinical Anatomy, School of Laboratory Medicine and Medical Sciences, College of Health Sciences, University of KwaZulu-Natal, Westville Campus, Private Bag X54001, Durban, 4000 South Africa; 2 Department of Neurosurgery, School of Clinical Medicine, College of Health Sciences, Nelson R Mandela School of Medicine, University of KwaZulu-Natal, Durban, South Africa; 3 Department of Radiology, Jackpersad and Partners Inc, Specialist Diagnostic Radiologists, Lenmed Ethekwini Hospital and Heart Centre, Durban, South Africa

**Keywords:** Anatomy, Medical research

## Abstract

The intracranial segment of the vertebral artery (VA) is the unique part of the artery where the two VAs join to form a single vascular channel, viz. the basilar artery. In addition to this typical description, anatomical variations have been described; the presence of anatomical variation has been associated with some pathological processes, neurological complications, and the risk of vascular diseases in the posterior circulatory territory. We evaluated the typical anatomical features and variations of the VA4 component of the VA in a South African population to provide useful data on the prevalence of variation and morphometry of the distal VA. The study is an observational, retrospective chart review of 554 consecutive South African patients (Black, Indian, and Caucasian) who had been examined with multidetector computed tomography angiography (MDCTA) from January 2009 to September 2019. We observed various anatomical variations in the VA4 segment of the VA. We report the incidence of VA hypoplasia, hypoplastic terminal VA, and atresia. Fenestration and duplicate posterior inferior cerebellar artery (PICA) origin were also observed. The left intracranial VA was significantly larger than the right. Our study shows that anatomical variation of the intracranial VA is common in the population studied, with a total prevalence of 36.5%. Understanding the patterns of anatomical variations of the VAs will contribute significantly to the interpretation of ischemic areas and diagnosis of various diseases in the posterior circulatory territory.

## Introduction

The vertebral artery (VA) emanates from the supero-posterior part of the subclavian artery and proceeds through the foramen transversarium of the sixth to first cervical vertebrae. The left and right VA penetrates the dura mater to enter the intracranial space through the foramen magnum, where they converge to form the basilar trunk at the pontomedullary junction^1^. Anatomically, the VA is divided into four segments. The first three segments (VA1, VA2, VA3) are the extracranial segments, extending from the origin to where they penetrate the dura mater. The fourth segment of the VA (VA4) is intracranial, extending from the foramen magnum to the point where the left and right VA anastomose to form the basilar trunk^1^. The geometry of the basilar trunk depends on the pattern of the bilateral VAs. When there is an asymmetry of bilateral VAs or other anatomical variations, the basilar trunk sometimes bends away from the midline^2^. Previously reported anatomical variations of the intracranial VAs include VA terminating as posterior inferior cerebellar artery (PICA), known as atresia, fenestration, asymmetry, and hypoplasia. Anatomical variations play a significant role in the clinical sequelae of an iatrogenic VA injury, which can vary widely^3^. For instance, damage to the dominant VA when the contralateral VA terminates as PICA can result in devastating complications since the dominant VA solely forms the basilar artery.

The presence of variation has been associated with some pathological processes, neurological complications, and the risk of vascular diseases in the posterior circulatory territory. For instance, atresia and hypoplasia have been associated with hypoperfusion of brain tissues and hemodynamic insufficiency, which may predispose to transient ischaemic attacks or acute brainstem ischaemic stroke^4,5^.

Reports on the prevalence of anatomical variations of the intracranial VA are scarce, with few previous reports in the Western population (Caucasians)^1^, the Asian population^4^, and the Turkish population^6^. Previous studies from the African continent used postmortem and cadaveric histological samples to report on average diameter and incidence of VA hypoplasia^7–9^. Advances in modern imaging technology that led to the establishment of multidetector computed tomography angiography (MDCTA) have made endovascular procedures popular. These procedures require a detailed understanding of typical anatomy and the extent of anatomical variations of the VAs. Consequently, a report on the prevalence of possible variant anatomy will help in the interpretation of radiographs, prevention of iatrogenic injuries, and contribute to the advancement of non-invasive surgical intervention.

In the present study, we assessed the typical anatomical features and variations of the V4 segment of the VA using MDCTA. We aimed to determine the dimensional characteristics and prevalence of anatomical variations of the intracranial VA in a South African population. Due to the multiracial composition of the South African population, in addition to the overall incidence of variation, we also report variations based on three racial groups: Black, Indian, and Caucasian South African. It is necessary to have correct and detailed information about the typical anatomy and prevalence of anatomical variations. Such information is essential before neurosurgery, endovascular and non-invasive procedures. The detailed information from this study will be useful in neurosurgery, anatomy, endovascular, and non-invasive procedures.

## Materials and methods

### Study population

This study is a retrospective observational review of 554 MDCTA images of South African patients. The patients underwent MDCTA for various reasons between January 2009 and September 2019. Images were obtained from the database of Lenmed Ethekwini Hospital and Heart Center, Durban, South Africa. The Biomedical Research Ethics Committee of the University of KwaZulu-Natal approved the study (Ethical No: BE 148/19) and waived the need for informed consent as this study utilized retrospective chart reviews. There was no patient contact, and no patient details were released from images. All methods were carried out in accordance with relevant guidelines and regulations. Exclusion criteria included MDCTA scans that showed no clarity of the VA's course, scans with motion artifacts or poor-quality imaging, and scans performed on foreign patients or obtained outside a hospital. The angiographies were from 307 males (55.4%) and 247 females (44.6%). The average age of the patients is reported as median (interquartile range〔IQR〕): 62 (23) (range: 10–99) years; 61 (23) for male patients and 62 (25) for female patients. Race was defined according to the guidelines outlined in the modern systems of racial classification in the Republic of South Africa^10^. The criteria used in the scheme of racial classification include skin colour and ancestry. The South African population is divided into four main racial groups: Caucasian, Black, Indian, and Coloured. Three population groups were included in the present study: Black 91 (16.4%), Indian 176 (31.8%), and Caucasian 287 (51.8%). According to the modern system of classification, a Caucasian was defined as a person of European descent. A Black individual was defined as a person having origins in any of indigenous Africa or Native group. An Indian individual was defined as a person of Asian descent^10^.

### MDCTA protocol

The imaging examination was performed on a 64-detector row 160-slice helical multidetector computed tomography scanner (Lightspeed CT, GE Healthcare Medical Systems, Milwaukee, WI, USA). In our standard MDCTA protocol for brain examinations, a scan coverage area from the aortic arch to the top of the brain in a supine position (headfirst) was adopted as a field of view (FOV). The scanning protocol was as follows: 120 kVp, 500 mAs, beam collimation 64 × 0.625 mm, speed 20.62 mm/rotation, gantry rotation time 0.35 s/rotation, the helical thickness of 0.625 mm, pitch 0.516:1, and reconstruction interval of 0.625 mm. Following the acquisition of the nonenhanced CT data, contrast-enhanced MDCTA was performed. During the procedure, a 20 Gauge needle (Pink Nexiva) was used to cannulate patients for IV access in the antecubital region to administer 60 mL of Ioversol (Optiray 350; Guerbet South Africa). This was followed by a 40 mL saline flush via a double power injector (Medex flowSens, Guerbet USA) into the patient’s antecubital vein (4 mL/s), and the scan delay was individually adapted using a bolus-tracking technique. First, a single nonenhanced low-dose scan at the upper neck level was obtained for the bolus tracking. With the start of contrast material administration, repeated low dose monitoring scans were obtained every second. Following the appearance of the first contrast in the aortic arch, the MDCTA was triggered automatically without delay. The region of interest was positioned at the aortic arch, and the threshold for the MDCTA was set as 150 Hounsfield Units. When the threshold was surpassed, helical scanning was automatically initiated.

### Imaging reconstruction

Postprocessing of three-dimensional images was performed using multiplanar reformation (MPR), maximum intensity projection (MIP), multiplanar reconstruction (MPR), and volume rendering (VR) algorithms. The volumetric MDCTA data sets were processed on Advanced Workstation 4.2 (GE Healthcare, Milwaukee, WI, USA). A series of 17 projection images at every 20° around the cephalocaudal axis were generated and transfer to the picture archiving communication system (PACS). The MDCTAs were performed for diagnostic purposes in the context of various cerebrovascular accidents or diseases. In some cases, the suspected diseases were not found on the MDCTA; thus, some materials in this study were derived from a healthy population.

### Analysis of anatomical variations and dimensions of the V4 segment

**Figure 1:**
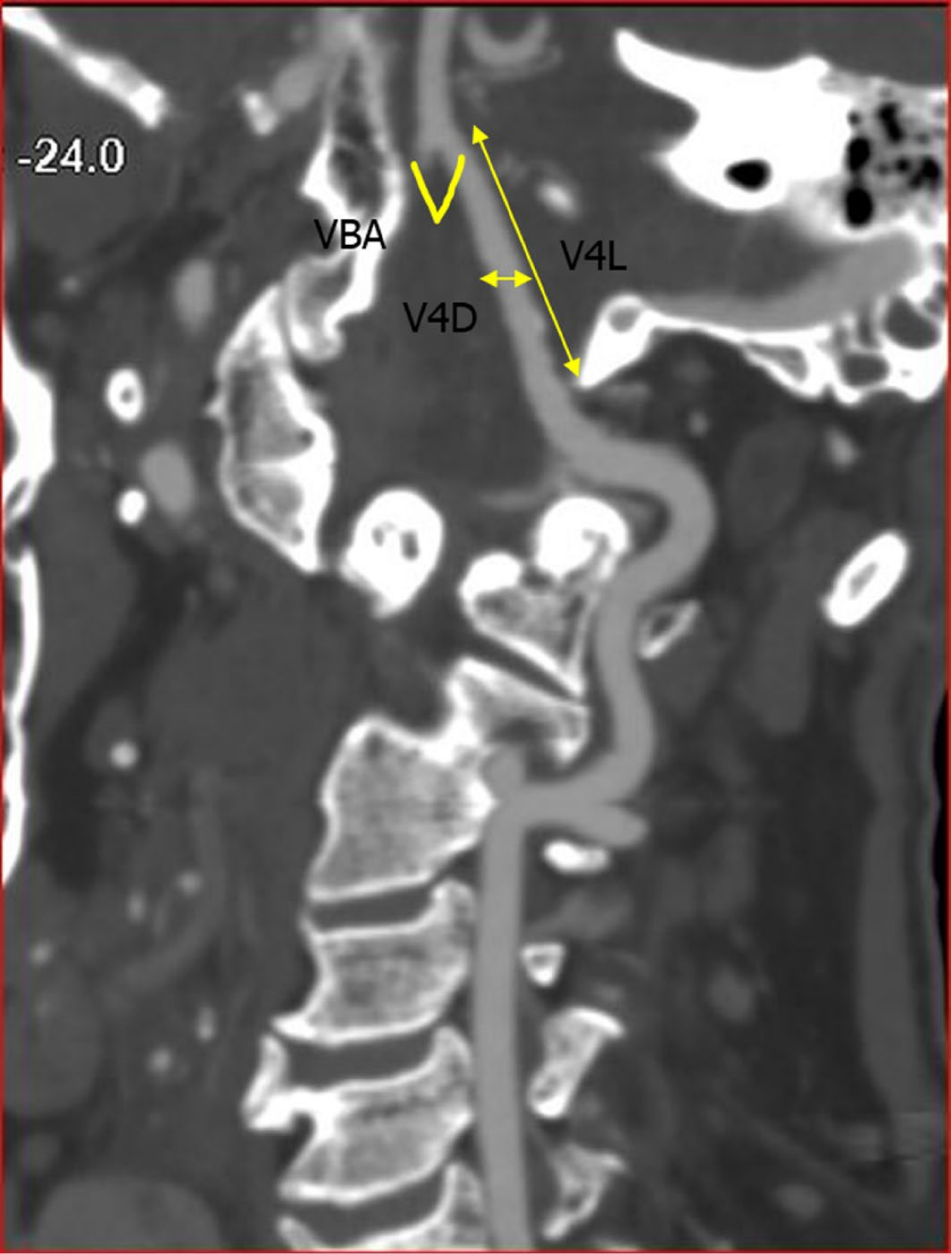
Oblique view of CTA image, showing the V3 and V4 segments of the VA. V4L length of the V4 segment; V4D Diameter of the V4 segment; VBA the angle at the vertebrobasilar junction.

The MDCTA images were analyzed using PACS tools. The images were examined for vascular variations by a neurosurgeon, a neuroradiologist, and an anatomist using the coronal and sagittal view and the 3D reconstructed images. Each MDCTA image was examined for the presence of anatomical variations. In atresia, the VA did not fuse with the contralateral VA but terminates as PICA. The hypoplastic terminal V4 segment was registered when the terminal portion divides into a PICA and a tiny branch that joins the contralateral dominant VA. Fenestration was registered when the VA split into two vessels, which later rejoined distally. The following parameters were measured on a coronal and oblique view of the MDCTA (Fig. 1): 1) the diameters were measured along the course of the VA at a distance of 11 mm cranial to the entrance of the VA into the foramen magnum, 2) the length of the VAs was measured from the foramen magnum to the point of union with the contralateral VA, and 3) the angle between the bilateral VA at the vertebrobasilar junction. We were unable to appropriately quantify the frequency of the PICA and the spinal arteries because visualization of branches (such as the PICA and spinal arteries) is usually beyond the limits of the MDCTA. A diameter of ≤ 2 mm was described as hypoplasia; we classified the VA as dominant if the diameter was larger than that of the contralateral side by any size difference according to the method provided by Ergun and co-authors^11^. When the bilateral VAs had a similar diameter, we referred to them as “equal” or “codominant.” Results were analyzed separately for the left and right sides.

### Statistical analysis

All data were analyzed using SPSS version 27 (SPSS Inc., Chicago, IL, USA). Categorical variables were analyzed using the chi-square test. A Kolmogorov–Smirnov test was used to assess the normal distribution of continuous data. Because the distribution of the data was not normal, nonparametric tests were used. The Kruskal–Wallis test followed by the Wilcoxon Signed-Rank test was used to detect significant differences in the obtained values. The interclass correlation coefficient was used to examine the reliability of measurements. All tests were performed at 95% confidence with a *p*-value of < 0.05.

### Ethics approval

The design was approved by the Institutional Review Board/Ethics Committee (Biomedical Research Ethics Committee of the University of KwaZulu-Natal with ethical No: BE 148/19).

## Results

Continuous and categorical data are presented as the median and IQR and percentage (N). The interclass correlation coefficient for intra-observer reliability testing was 92% for V4 length, 93% for diameter, and 96% for the angle at the VBJ. For inter-observer reliability testing, the intraclass correlation was 85% for V4 length and diameter; 87% for the angle at the VBJ.

### Variation in morphology

**Figure 2:**
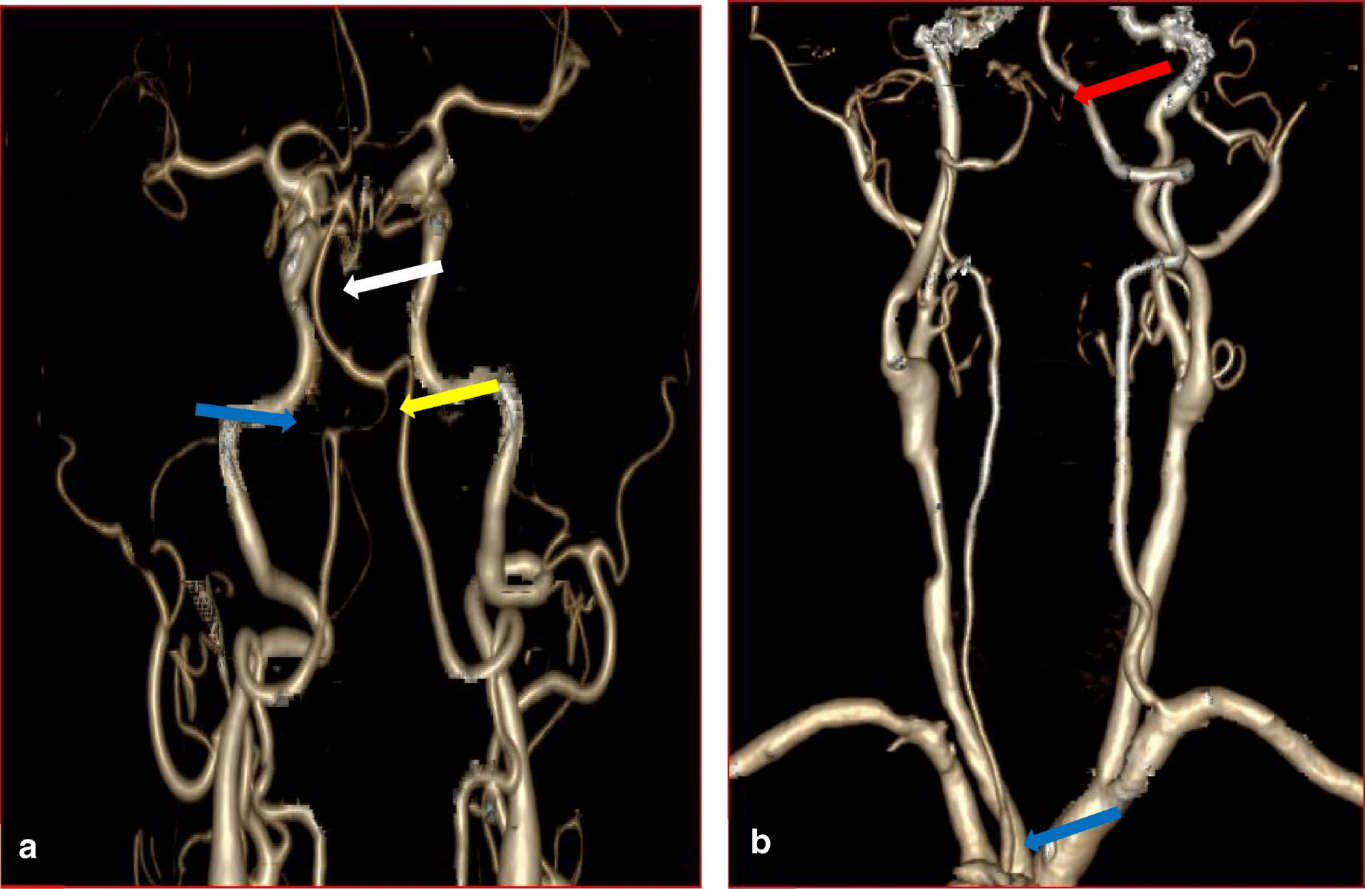
3D-CTA reconstructed images showing the vertebral, the subclavian, and the carotid arteries. (**a**) The blue arrow indicates PICA. The yellow arrow indicates hypoplastic terminal VA while the white arrow indicates the bending basilar artery. (**b**) The blue arrow illustrates the origin of the left VA from the arch of the aorta, and the red arrow shows the termination of ipsilateral VA as PICA.

**Figure 3:**
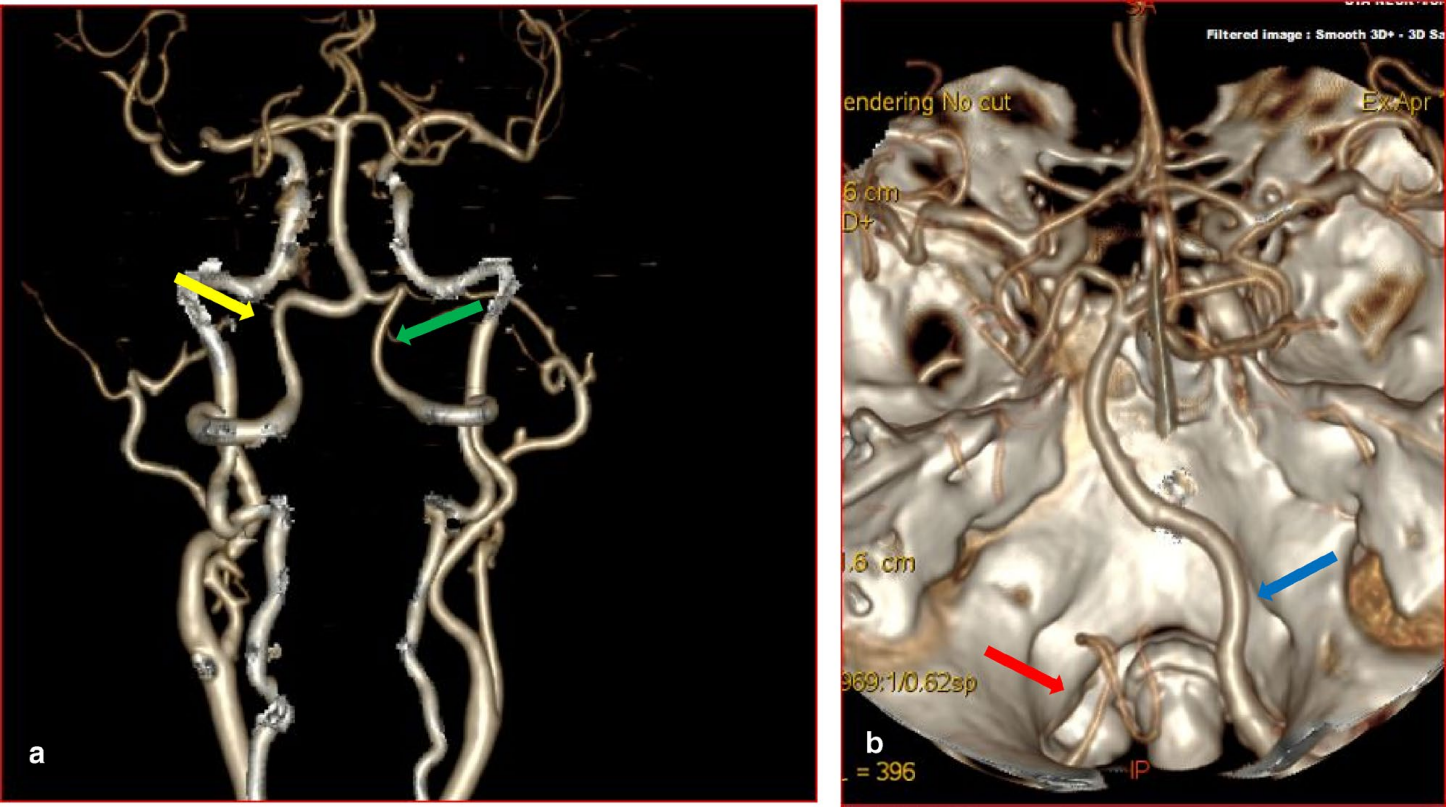
3D-CTA reconstructed images showing the vertebral, the basilar arteries, and the Circle of Willis. (**a**) The yellow arrow illustrates the left dominant VA. The green arrow indicates hypoplastic right VA. (**b**) The red arrow illustrates termination of left VA as PICA. The blue arrow indicates dominant right VA.

**Figure 4:**
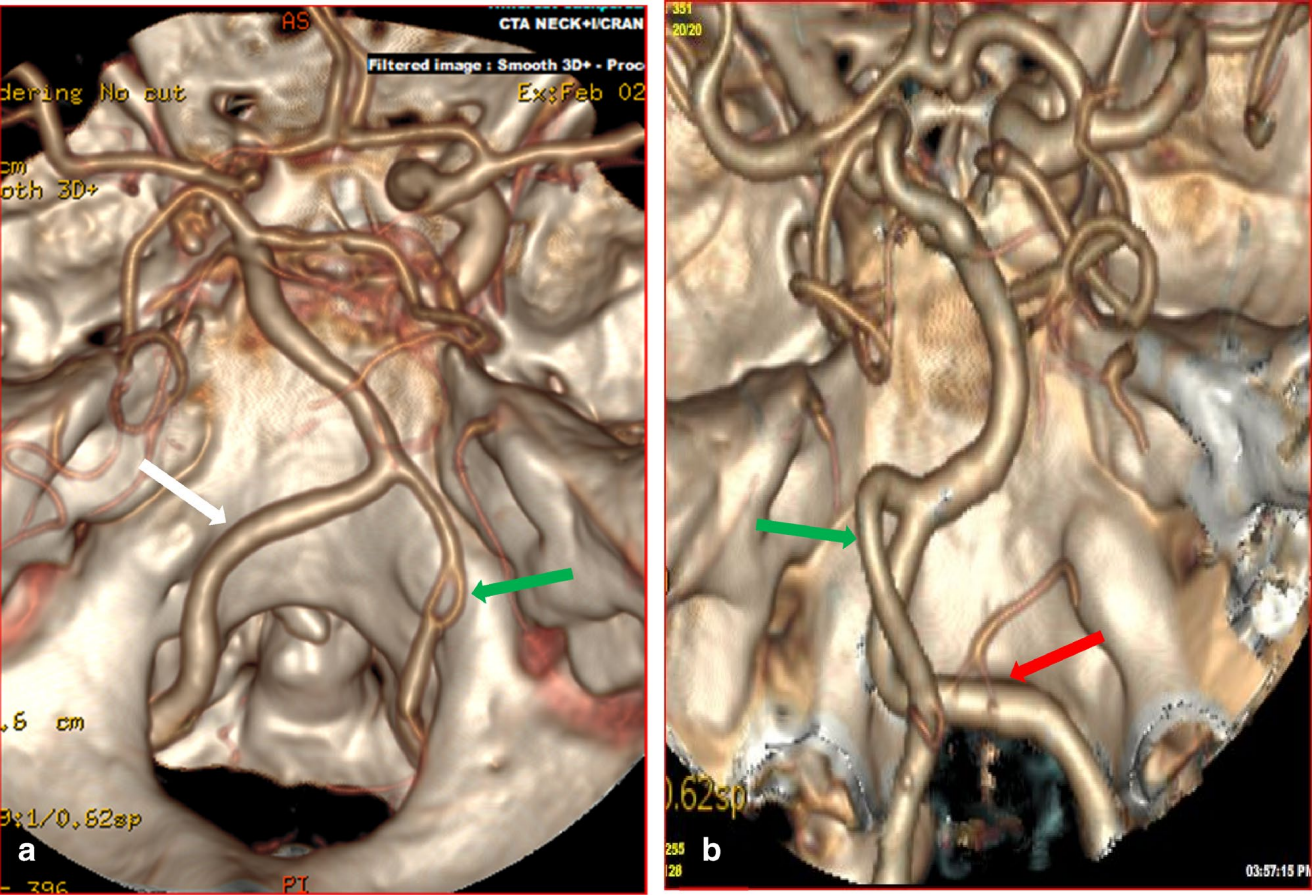
3D-CTA reconstructed images showing the vertebral, the basilar arteries, and the Circle of Willis. (**a**) The green arrow indicates fenestration of right intracranial VA while the white arrow indicates the left VA. (**b**) The red arrow illustrates duplicate PICA origin, while the green arrow indicates the left VA.

We observed the following variations of the intracranial segment: (1) The hypoplastic terminal VA (Fig. 2a) and hypoplasia of VA (Fig. 3a). (2) VA terminating as PICA (Atresia) (Figs. 2b and 3b). (3) Fenestration (One was observed at the right intracranial VA (Fig. 4a), while the other was observed at the vertebrobasilar junction). (4) Duplicate origin of the PICA (Fig. 4b). The incidence of these variations is summarized in Table 1. The incidence of VA hypoplasia is significantly high in Caucasian, followed by Indian on the right (*p* = 0.01). There was no significant difference across the races on the left (*p* = 0.61). Also, there was no significant racial difference in the incidence of hypoplastic terminal VA (*p* = 0.26) and atresia (*p* = 0.54). The incidence of variation across the races is summarized in Table 2. Because visualization of branches of the intracranial VA (PICA and spinal arteries) is usually beyond the limits of MDCTA, the frequency of PICA on the left, right, and bilateral PICA in the present study is low (15.7%, 13.9%, and 14.8%, respectively). We also observed bilateral and unilateral double PICA in 5 patients. Therefore, we cannot appropriately quantify the frequency of the PICA in all the VAs.

**Table 1:** Incidence of anatomical variations at the intracranial segment (V4) of the VA diagnosed by MDCTA.

Type of Variation	Total number of patients (incidence%)	Left/Right/Bilateral	Male/Female
Hypoplasia	89 (16.1)	36/46/7	56/33
Hypoplastic terminal VA	73 (13.2)	37/36/0	46/27
VA terminating as PICA (Atresia)	37 (6.7)	15/22/0	24/13
Fenestration	2 (0.4)	0/1/0	1/1
Duplicate PICA origin	1 (0.2)	0/1/0	0/1

**Table 2:** Incidence of anatomical variations at the intracranial segment (V4) of the VA grouped according to race in South African patients.

Type of Variation	Total number of patients (incidence%)	Race		
		Black	Indian	White
		Left/Right/Bilateral	Left/Right/Bilateral	Left/Right/Bilateral
Hypoplasia	89 (16.1)	5/5/1	10/19/3	21/22/3
Hypoplastic terminal VA	73 (13.2)	9/3/0	8/15/0	20/18/0
VA terminating as PICA (Atresia)	37 (6.7)	1/2/0	4/9/0	10/11/0
Fenestration	2 (0.4)	0	0	0/1/1
Duplicate PICA origin	1 (0.2)	0	0	0/1/0

### Morphometric analysis of the intracranial vertebral arteries

#### Diameter

The average diameter of the left VA (3.17 (0.62) mm) was similar to that of the right VA (3.17 (0.7) mm). However, the Wilcoxon Signed-Rank test showed a significant difference (*p* < 0.001). This is because the Wilcoxon Signed-Rank test is a rank-sum test and not a median test. The sum of positive Ranks of the left VA was significantly greater than that of the right VA. We observed a left pattern of dominance in 45.3% (251/554) patients; the right side was dominant in 32.7% (181/554) patients. The left and right VAs was equal in diameter in 15.3% (85/554) patients. Concerning the racial groups, no significant differences were observed (Right VA, *p* = 0.567; Left VA, *p* = 0.180). The group diameters are summarized in Table 3. For gender, the diameters are summarized in Table 4. There were no significant gender differences in VA diameter (Right VA, *p* = 0.528; Left VA, *p* = 0.274).

**Table 3:** Diameter and length of the vertebral artery V4 segment grouped according to race and laterality in South African patients.

	Black		Indian		White	
	Left	Right	Left	Right	Left	Right
V4 Diameter	3.17 (0.7)	3.17 (0.7)	3.17 (0.69)	3.17 (0.7)	3.17 (0.62)	3.17 (0.68)
V4 Length	32.74 (9.13)	30.71 (9.15)	32.20 (7.74)	30.86 (8.6)	32.38 (6.87)	31.61 (6.34)

Results are reported as median (*IQR* interquartile range) mm.

**Table 4:** Diameter and length of the vertebral artery V4 segment grouped according to gender and laterality in South African patients.

	Male		Female	
	Left	Right	Left	Right
V4 Diameter	3.17 (0.62)	3.17 (0.7)	3.17 (0.62)	3.17 (0.62)
V4 Length	32.29 (7.34)	31.59 (6.92)	32.38 (6.68)	31.48 (7.69)

Results are reported as median (*IQR* interquartile range) mm.

#### Length

The length of the left (32.36 (7.18) mm) intracranial VA was significantly greater than the right (31.50 (7.22) mm). Within the racial groups, there were no significant differences (Right VA, *p* = 0.386; Left VA, *p* = 0.708). The average length and laterality of the VA across the racial groups are summarized in Table 3. There were no significant gender differences in the length of the VA (Right VA, *p* = 0.665; Left VA, *p* = 0.615). The results are summarized in Table 4.

#### The angle at the vertebrobasilar junction

The angle at the vertebrobasilar junction was 46° (18°). Within the racial groups, the average angle in Black patients (51° (22°)) was significantly larger than in Caucasian (47° (18°) *p* = 0.037) and Indian (42° (16°) *p* = 0.000) patients. A significant difference was also observed between the Caucasian and Indian (*p* = 0.010) patients. There were no significant gender differences (*p* = 0.103).

## Discussion

Our study shows that MDCTA made it possible to evaluate anatomical variations of the intracranial VA. We found that variation is common in the population studied, with a total prevalence of 36.5%. The most frequently observed is VA hypoplasia. The incidence of hypoplasia in the present study (8.3% on the right and 6.5% on the left) is similar to the report of Ergun et al.^11^. These authors defined VA hypoplasia using diameter criteria of ≤ 2 mm and reported an incidence of 7.1% on the right and 9.4% on the left among 254 patients in their angiographic series^11^. By contrast, Songur and co-authors in their autopsy study reported a relatively high incidence of 20.2% on the right, 14.4% on the left, and 4.3% bilaterally using a similar definition of VA hypoplasia^6^. Sometimes it is challenging to compare data from different populations and research groups due to the differences in study modalities, distribution of data, and average diameter. According to a recent report, an individual's VA diameter may depend on anthropometric parameters such as height^12^. All these factors may contribute to the wide range of differences reported in the literature. VA hypoplasia is a congenital anatomical variation that has been previously described with a cut-off diameter between 2.0 and 3.0 mm^13^. During embryogenesis, the VA is formed from multiple longitudinal anastomoses between adjacent cervical intersegmental arteries^14,15^. The mechanism of the formation of VA hypoplasia is not precise. However, some authors hypothesized delayed development of the vertebrobasilar artery as the major cause of VA hypoplasia^16^.

The reduced diameter of hypoplastic VA has been associated with an increased probability of spontaneous dissection^17^ and ipsilateral PICA and lateral medullary infarctions due to suspected atherosclerosis as a result of abnormal hemodynamics^18^. Recently, VA hypoplasia has been associated with an aneurysm of the contralateral dominant VA, most especially at the site of PICA origin^19^. Knowledge of pathologies associated with VA hypoplasia can provide clues and help diagnose pathological processes in the posterior circulatory territory.

In addition to hypoplasia, we also noticed that the hypoplastic terminal portion of the unilateral intracranial VA is a common anatomical variant of the studied population. The VA seems to divide at a spot along its courses to a PICA branch and a tiny branch that joins the contralateral VA. Pekcevik and co-author proposed another terminology for this type of variation; VA continued as PICA^20^. In our own opinion, this suggested anatomical term can be confused with VA terminating as PICA or VA ending as PICA (atresia). We suggest that this variant anatomy can be simply described as hypoplastic terminal VA.

In our series, the percentage of patients having VA atresia is 6.7%. Prevalence of VA atresia has previously been reported as up to 9%^4,21^. Our results were in accordance with the range of the reported prevalence but most similar to that reported by Liu et al. (6.3%)^4^. Clinically, VA atresia has been previously linked to rotational vertebral artery syndrome (RVAS)^22^ and bow hunter’s syndrome^23,24^, which may result from compression of this variant vessel. Similar to VA hypoplasia, the embryological basis of VA atresia is unclear. The first of the seven cervical intersegmental arteries that formed the VA was designated as the proatlantal intersegmental artery, while the seventh intersegmental artery forms the proximal part of the subclavian artery and point of origin of the VA^15^. Each of these intersegmental arteries is a potential site of arterial agenesis that could result in anatomical variation^25^. Since the point of origin of the VA is at the seventh intersegmental artery, the intracranial VA may have developed from the proatlantal intersegmental artery. Complications during the process of fusion and anastomosis of the proatlantal and other cervical intersegmental arteries could result in anatomical variations of the distal VAs which may include atresia.

We also observed fenestration at the right intracranial VA in one patient and the proximal part of the basilar artery in another patient. Our observation is similar to the report of Dzierzanowski et al., which reported two fenestrations in the Caucasians^1^. Fenestration of the vertebrobasilar artery is a congenital anomaly that involves lumina division of an artery with a single origin into two separate channels that later reunite distally. Embryologically, the VA and the basilar artery develop from different primitive vessels. The VA is formed from the cervical intersegmental arteries, while the basilar artery develops from the longitudinal neural arteries. As a result of these, fenestration at the V4 segment of the VA is due to the absence of obliterations of two intersegmental vessels that fused^25^. Fenestration of the proximal basilar occurs due to partial failure or incomplete fusion of the longitudinal neural arteries and regression of the bridging arteries connecting the longitudinal arteries^26^. Fenestration may predispose to aneurysm around the fenestrated portion of the artery^6,20^, and it has also been previously associated with unexplained subarachnoid hemorrhage^27^. In addition to the associated pathologies, knowledge of this variation is essential in clinical diagnosis as fenestration may be misinterpreted as an aneurysm or a dissection on magnetic resonance imaging^20^.

In the present study, duplicate PICA origin was registered in one of the patients. It is important to note that duplicate PICA origin is different from the duplication of the PICA. In the duplicate origin, the PICA has two separate origins that later converge distally in the course of the artery. Whereas in duplication of the PICA, there is no distal arterial convergence^28^: each artery courses separately. Duplicate PICA origin is a rare congenital anatomical variation of the PICA with a prevalence of roughly 1.45% previously reported in the Western population (Caucasian and Asian)^28^. Clinically, duplicate PICA origin has been previously reported to highly predispose to intracranial aneurysm formation with an associated incidence between 50 and 71%^28,29^. Embryologically, Lesley and co-authors hypothesized that duplicate PICA origin might be a manifestation of underlying deficient vascular developmental disorganization, which may upraise the tendency toward formation of an intracranial aneurysm^28^. Other authors suggested that this anatomical variation may have resulted from variation in the persistence of standard anastomosis between the lateral spinal artery and the PICA^30^. Considering the unique embryogenesis, adequate perfusion of the regions supplied by the PICA may rely on flow from both origins^29^. Since duplicate PICA origin is an uncommon variation with few previous reports, it should not be overlooked when evaluating the diagnosis and surgical intervention images.

The average diameter and length of the VA in our result is consistent with the previous report on a South American population (Diameter Left- 3.12 ± 0.85 mm, Right- 2.94 ± 0.77 mm; Length Left- 33.86 ± 5.59 mm, Right- 32.47 ± 4.8 mm)^31^ based on autopsy samples and another angiographic study of the Caucasians (Diameter Left- 3.16 ± 0.63 mm, Right- 2.78 ± 0.44 mm; Length Left- 31.51 ± 6.51 mm Right- 24.25 ± 6.76 mm)^1^. We observed a significantly larger diameter on the left than the right VA, comparable to the previous reports mentioned above. Interestingly, there was no significant difference across the racial groups and gender in our series. By contrast, a previous histological study of a South African population (Witwatersrand region) reported an average diameter (Left- 2.68 ± 0.86 mm, Right- 2.53 ± 0.75 mm)^7^ that was lower than the present study. The differences in the study modalities (MDCTA vs. cadaveric) may be responsible for the contrariety noticed in the results. Tissue shrinkage associated with histological tissue processing may be the reason for the reduced diameter.

We described the pattern of dominance using the criterion of any size difference between the left and right VA; 45.3% showed left dominance, 32.7% showed right dominance, and 15.3% showed codominance. Using a similar criterion, Ozdemir et al. reported similar results of left dominance in 64% of patients and right dominance in 31% of patients^32^. In contrast, Ergun and co-authors reported right VA dominance in 49.5% and left dominance in 47.2% of patients using a similar criterion as described above^11^. Our result shows that most of the patients have left dominant VAs. Noticeably, we observed more VA hypoplasia and atresia on the right. Knowledge of the dominant VA is required for some endovascular procedures. It is also important to preserve the dominant VA since they are likely to predominates the basilar artery. This information is vital to reduce the risk of neurological symptoms that may results from iatrogenic injury.

The angle at the vertebrobasilar junction in the present study is comparable with the report of Songur et al. (52.2 ± 18.2°)^6^. On the contrary, other authors reported a larger mean angle (85.45 ± 10.76°)^1^. The disparity may have resulted from the confluence of the bilateral VA, which can either be a sharp or blunt edge depending on the pattern and frequency of asymmetry. In atresia, the VA did not fuse with the contralateral VA but terminated as PICA. The contralateral VA solely proceeds to form the basilar artery. In the case of hypoplastic terminal VA, the contralateral VA predominates the basilar artery with little contribution from the tapering end of the hypoplastic terminal VA. In addition to asymmetry, these two conditions can also cause the basilar artery to bend from the midline (also known as bending basilar)^33^. Deviation and prominence of a vessel, such as bending basilar due to dominance of one of the VAs, may cause compression of cranial nerves^20^. Furthermore, it is essential to consider the geometry of the vertebrobasilar junction while planning for surgical interventions in this region. This region is of particular interest to neurosurgeons and radiologists due to various interventional neuroradiological procedures conduct in the area to treat vascular diseases such as arterial dissections, aneurysms, arteriovenous malformations, dural fistula, or repair of an occlusive disease^34^.

## Conclusion

Our study shows that anatomical variation of the intracranial VA is common in the population studied, with a total prevalence of 36.5%. Hypoplasia and hypoplastic terminal VA being the most frequent. Understanding the patterns of anatomical variations of the VAs will contribute significantly to the interpretation of ischemic areas and diagnosis of various diseases in the posterior circulatory territory.

## Data Availability

Available on request.

## References

[1] Dzierzanowski J (2017). Intracranial region of the vertebral artery: morphometric study in the context of clinical usefulness. Folia Morphol. (Warsz.).

[2] Hong J, M. (2009). Vertebral artery dominance contributes to basilar artery curvature and peri-vertebrobasilar junctional infarcts. J. Neurol. Neurosurg. Psychiatr..

[3] Schroeder GD, Hsu WK (2013). Vertebral artery injuries in cervical spine surgery. Surg. Neurol. Int..

[4] Liu I-W (2017). Vertebral artery terminating in posterior inferior cerebellar artery: A normal variation with clinical significance. PLoS One.

[5] Chen Y-Y, Chao A-C, Hsu H-Y, Chung C-P, Hu H-H (2010). Vertebral artery hypoplasia is associated with a decrease in net vertebral flow volume. Ultrasound Med. Biol..

[6] Songur A (2008). Variations in the intracranial vertebrobasilar system. Surg. Radiol. Anat..

[7] Mitchell J (2004). Differences between left and right suboccipital and intracranial vertebral artery dimensions: an influence on blood flow to the hindbrain?. Physiother. Res. Int..

[8] Mitchell J, McKay A (1995). Comparison of left and right vertebral artery intracranial diameters. Anat. Rec..

[9] Ogengo J, Olabu B, Sinkeet R, Ogengo NM, Elbusaid H (2014). Vertebral artery hypoplasia in a Black Kenyan population. J. Int. Sch. Res. Not..

[10] Khalfani AK, Zuberi T (2001). Racial classification and the modern census in South Africa, 1911–1996. Race Soc..

[11] Ergun O, Tatar IG, Birgi E, Hekimoglu B (2016). Evaluation of vertebral artery dominance, hypoplasia and variations in the origin: angiographic study in 254 patients. Folia Morphol..

[12] Gaigalaite V (2016). Association between vertebral artery hypoplasia and posterior circulation stroke. J. BMC Neurol..

[13] Katsanos AH, Kosmidou M, Kyritsis AP, Giannopoulos S (2013). Is vertebral artery hypoplasia a predisposing factor for posterior circulation cerebral ischemic events? A comprehensive review. Eur. Neurol..

[14] Luh G, Dean B, Tomsick T, Wallace R (1999). The persistent fetal carotid-vertebrobasilar anastomoses. AJR Am. J. Roentgenol.

[15] Padget DH (1948). The development of the cranial arteries in the human embryo. J. Contrib. Embryol..

[16] Kim C, Sohn J-H, Choi H-C (2017). Are the anomalous vertebral arteries more hypoplastic?: retrospective linear mixed model approach. BMC Neurol..

[17] Zhou M (2015). Vertebral artery hypoplasia and vertebral artery dissection a hospital-based cohort study. Neurology.

[18] Chuang Y-M, Huang Y-C, Hu H-H, Yang C-Y (2006). Toward a further elucidation: role of vertebral artery hypoplasia in acute ischemic stroke. J. Eur. Neurol..

[19] Harati A (2019). Association between vertebral artery hypoplasia and vertebral artery aneurysms: A case-control study. J. Clin. Neurosci..

[20] Pekcevik Y, Pekcevik R (2014). Variations of the cerebellar arteries at CT angiography. Surg. Radiol. Anat..

[21] Ohkura K (2014). Vertebral artery variations in thoracic aortic patients. Eur. J. Cardiothorac. Surg..

[22] Noh Y, Kwon O-K, Kim H-J, Kim JS (2011). Rotational vertebral artery syndrome due to compression of nondominant vertebral artery terminating in posterior inferior cerebellar artery. J. Neurol..

[23] Yeh J-F (2005). A case of bow hunter’s stroke caused by non-dominant vertebral artery. Acta Neurol. Taiwan..

[24] Miao H-L, Zhang D-Y, Wang T, Jiao X-T, Jiao L-Q (2020). Clinical Importance of the Posterior Inferior Cerebellar Artery: A Review of the Literature. Int. J. Med. Sci..

[25] George B, Bruneau M, Spetzler RF (2013). Pathology and surgery around the vertebral artery.

[26] Zhu D-Y (2016). Treatment of fenestrated vertebrobasilar junction-related aneurysms with endovascular techniques. J. Clin. Neurosci..

[27] Hudák I, Lenzsér G, Lunenkova V, Dóczi T (2013). Cerebral arterial fenestrations: a common phenomenon in unexplained subarachnoid haemorrhage. Acta Neurochir. (Wien.).

[28] Lesley WS, Rajab MH, Case RS (2007). Double origin of the posterior inferior cerebellar artery: association with intracranial aneurysm on catheter angiography. Am. J. Roentgenol..

[29] Silva MA, See AP, Aziz-Sultan MA, Patel NJ (2017). Surgical treatment of a double origin posterior inferior cerebellar artery aneurysm and insights from embryology: case report and literature review. Oper. Neurosurg..

[30] Lasjaunias P, Vallee B, Person H, Ter Brugge K, Chiu M (1985). The lateral spinal artery of the upper cervical spinal cord: anatomy, normal variations, and angiographic aspects. J. Neurosurg..

[31] Ballesteros L, Forero P, Quintero I (2013). Morphological expression of the anterior spinal artery and the intracranial segment of the vertebral artery: a direct anatomic study. Rom. J. Morphol. Embryol..

[32] Ozdemir S, Yildiz C, Cankur N (2002). Evaluation of vertebral artery system in a healthy population by using colour duplex Doppler ultrasonography. Med School J Uludag.

[33] Meng X, Ding W, Wu X, Di P (2018). Clinical investigation and characterization of vertebrobasilar dolichoectasia and vertebral artery dominance. Discov. Med..

[34] Mercier P (2008). Vascular microanatomy of the pontomedullary junction, posterior inferior cerebellar arteries, and the lateral spinal arteries. Interv. Neuroradiol..

